# Supplementary material for: Hybrid Models and Biological Model Reduction with PyDSTool
Source: PLoS Comput Biol. 2012 Aug 9;8(8):e1002628. doi: 10.1371/journal.pcbi.1002628 (PMC3415397; doi:10.1371/journal.pcbi.1002628)
Supplement: Text S4 — Complete source code for the PyDSTool package (version 0.88.120504). Includes API documentation and help files linking to web pages. This file is identical to the current public release on Sourceforge.net. (ZIP) [file pcbi.1002628.s004.zip › PyDSTool/html/PyDSTool.Toolbox.FSM-module.html]

xml version="1.0" encoding="ascii"?


PyDSTool.Toolbox.FSM


| Home | Trees | Indices | Help | PyDSTool |
| --- | --- | --- | --- | --- |

Package PyDSTool ::
Package Toolbox ::
Module FSM

[hide private]

[frames] | no frames]

# Module FSM

source code

```
Finite State Machines.

By Noah Spurrier (2002) and other contributors,
from http://code.activestate.com/recipes/146262-finite-state-machine-fsm/

This module implements a Finite State Machine (FSM). In addition to state
this FSM also maintains a user defined "memory". So this FSM can be used as a
Push-down Automata (PDA) since a PDA is a FSM + memory.

The following describes how the FSM works, but you will probably also need to
see the example function to understand how the FSM is used in practice.

You define an FSM by building tables of transitions. For a given input symbol
the process() method uses these tables to decide what action to call and what
the next state will be. The FSM has a table of transitions that associate:

        (input_symbol, current_state) --> (action, next_state)

Where "action" is a function you define. The symbols and states can be any
objects. You use the add_transition() and add_transition_list() methods to add
to the transition table. The FSM also has a table of transitions that
associate:

        (current_state) --> (action, next_state)

You use the add_transition_any() method to add to this transition table. The
FSM also has one default transition that is not associated with any specific
input_symbol or state. You use the set_default_transition() method to set the
default transition.

When an action function is called it is passed a reference to the FSM. The
action function may then access attributes of the FSM such as input_symbol,
current_state, or "memory". The "memory" attribute can be any object that you
want to pass along to the action functions. It is not used by the FSM itself.
For parsing you would typically pass a list to be used as a stack.

The processing sequence is as follows. The process() method is given an
input_symbol to process. The FSM will search the table of transitions that
associate:

        (input_symbol, current_state) --> (action, next_state)

If the pair (input_symbol, current_state) is found then process() will call the
associated action function and then set the current state to the next_state.

If the FSM cannot find a match for (input_symbol, current_state) it will then
search the table of transitions that associate:

        (current_state) --> (action, next_state)

If the current_state is found then the process() method will call the
associated action function and then set the current state to the next_state.
Notice that this table lacks an input_symbol. It lets you define transitions
for a current_state and ANY input_symbol. Hence, it is called the "any" table.
Remember, it is always checked after first searching the table for a specific
(input_symbol, current_state).

For the case where the FSM did not match either of the previous two cases the
FSM will try to use the default transition. If the default transition is
defined then the process() method will call the associated action function and
then set the current state to the next_state. This lets you define a default
transition as a catch-all case. You can think of it as an exception handler.
There can be only one default transition.

Finally, if none of the previous cases are defined for an input_symbol and
current_state then the FSM will raise an exception. This may be desirable, but
you can always prevent this just by defining a default transition.

Noah Spurrier 20020822
```


|  |  |
| --- | --- |
| Classes | [hide private] |

ExceptionFSM  
This is the FSM Exception class.

FSM  
This is a Finite State Machine (FSM).

ObjFSM  
A subclass of FSM where input\_symbol may be any kind of object,
even an unhashable one.

| Home | Trees | Indices | Help | PyDSTool |
| --- | --- | --- | --- | --- |

|  |  |
| --- | --- |
| Generated by Epydoc 3.0.1 on Fri May 4 15:24:04 2012 | http://epydoc.sourceforge.net |
